# The Value of Contrast-Enhanced Ultrasound Classification in Diagnosis of Pancreatic Cystic Lesions

**DOI:** 10.1155/2019/5698140

**Published:** 2019-10-13

**Authors:** Yixi Wang, Yanjie Wang, Zhihui Fan, Jun Shan, Kun Yan

**Affiliations:** ^1^Key Laboratory of Carcinogenesis and Translational Research (Ministry of Education), Department of Ultrasound, Peking University Cancer Hospital and Institute, No. 52, Fucheng Road, Haidian District, Beijing 100142, China; ^2^Key Laboratory of Carcinogenesis and Translational Research (Ministry of Education), Department of Radiology, Peking University Cancer Hospital and Institute, No. 52, Fucheng Road, Haidian District, Beijing 100142, China

## Abstract

**Objective:**

To compare the consistency of contrast-enhanced ultrasound (CEUS) classification results with magnetic resonance imaging (MRI) and to investigate the diagnostic value of CEUS classification in pancreatic cystic lesions.

**Methods:**

84 cases of pancreatic cystic lesions were enrolled in this study. According to the CEUS classification methods of previous study in our center, all the lesions were classified into four types: type I, unilocular cysts; type II, microcystic lesions; type III, macrocystic lesions; and type IV, cystic lesions with enhanced solid components. The consistency of CEUS and MRI typing results was analysed. Among the 84 cases, 45 cases had pathological results. The CEUS results were compared with the pathological results, and the diagnostic value of CEUS classification in diagnosing pancreatic cystic lesions was explored.

**Results:**

Among the 84 cases, CEUS diagnosed 8 cases of type I, 24 of type II, 8 of type III, and 45 of type IV. MRI diagnosed 10 cases of type I, 25 of type II, 7 of type III, and 43 of type IV. The classification typing results of CEUS were highly consistent with that of enhanced MRI (kappa value: 0.852). Among the 45 cases with pathological results, the diagnostic accuracy of each type was 91.1%, 95.6%, 93.3%, and 88.9%. The accuracy of CEUS and MRI in diagnosing pancreatic cystic lesions was 75.56% (34/45) and 80% (36/45), respectively. The diagnostic accuracy of CEUS had no significant difference from that of MRI (*P*=0.687).

**Conclusion:**

The classification results by CEUS and MRI are in excellent agreement. The classification of pancreatic cystic lesions by CEUS is significantly helpful for clinical diagnosis.

## 1. Introduction

With the development of medical imaging technology, the detection rate of pancreatic cystic lesions has gradually increased. According to histopathology, pancreatic cystic lesions can be divided into nonneoplastic cysts and neoplastic lesions roughly. Furthermore, there are multiple pathological types in tumor lesions, for instance, serous cystic neoplasms (SCNs), mucinous cystic neoplasms (MCNs), intraductal papillary mucinous tumors (IPMNs), pancreatic carcinoma with cystic degeneration, and other types. Because of the great difference in the treatment and prognosis of various types, preoperative identification of the type of pancreatic cystic lesions has a great guiding effect. At present, there are no unified internationally recognized imaging classification methods for pancreatic cystic lesions. Similar to the Bosniak classification of renal cysts [1], prestudy of our center [2] had classified pancreatic cystic lesions into 4 types based on the differences in anatomical features and contrast-enhanced ultrasound (CEUS) characteristics: type I, unilocular cysts; type II, microcystic lesions; type III, macrocystic lesions; and type IV, cystic lesions with solid components or irregular thickened cystic wall. Sahani et al. [3] had proposed a similar classification based on magnetic resonance imaging (MRI), and he thought the classification could narrow the differential diagnosis of pancreatic cystic lesions. This study aimed to compare CEUS results with MRI results and investigate the diagnostic value of CEUS classification for pancreatic cystic lesions.

## 2. Materials and Methods

### 2.1. Subjects

From December 2013 to March 2019, 84 patients with pancreatic cystic lesions who had underwent CEUS and MRI in our hospital simultaneously (with interval no more than four weeks) were enrolled in this study, including 23 males and 61 females, aged 17–76 years, average age of 52.64 ± 15.02 years. There were a total of 84 lesions, and the maximum diameter of the lesions ranged from 1.2 to 14.2 cm, with an average of 4.3 ± 2.3 cm.

Institutional review board approval was obtained, and informed consents were signed by all the patients before the study.

### 2.2. CEUS Techniques

The GE Logiq E9 ultrasound machine was used with a probe frequency of 2.0 MHz. The mechanical index was 0.08–0.12. We chose SonoVue (Bracco Milan, Italy) as the CEUS contrast agent. The Sonovue lyophilized powder was dissolved in 5 ml of saline, shook, and then mixed into a suspension for use. Each contrast, 1.5 ml of suspension, was injected rapidly through the antecubital vein, followed by a 5 ml saline flush. The CEUS mode was entered after injection, and the perfusion process of the lesion, septa, or solid components was observed in real time. The dynamic images were stored after examination.

### 2.3. Enhanced MRI

MRI examination was performed using the magnetic resonance imaging system Discovery MR750 3.0T (GE Medical Systems, Discovery, USA). Imaging sequences include axial T2-weighted sequences: TR/TE = 3000 ms/85 ms, layer thickness 4 mm, interval 0.4 mm, excitation times 8 times; axial LAVA-Flex dynamic enhanced sequence scanning: TR/TE = 6.0 ms/2.1 ms, flip angle 10°, layer thickness 4∼5 mm, interval 0 mm; and axial T2WI anti-lipid sequence scanning: TR/TE = 6500 ms/102 ms, layer thickness 7 mm, interval 1 mm, excitation times 4 times. A 15 ml injection of gadopentetate dimeglumine (Gd-DTPA) (0.2 mmol per kilogram of body weight) was injected via elbow vein using a high pressure syringe, followed by 20 mL of saline. The four enhanced scanning phases are as follows: arterial (15–20 s), parenchymal (40–50 s), portal venous phase (70–80 s), and delayed phase (140–150 s).

### 2.4. Classification of Pancreatic Cystic Lesions by CEUS and MRI

CEUS images were analysed by two ultrasound physicians with at least 5 years of experience in CEUS individually. They did not know any information about the pathological results, clinical diagnosis, or other imaging data beforehand. When there was disagreement, the conclusion was reached after discussion.

MRI images were analysed by experienced imaging doctors, and pathological results and clinical diagnosis were not known in advance.

**Figure 1:**
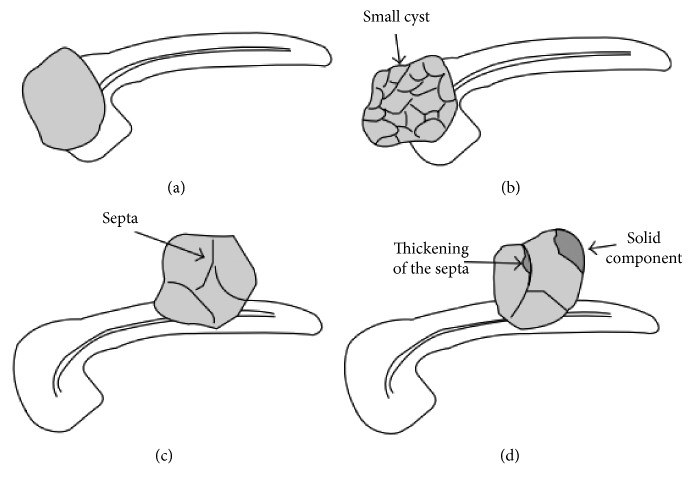
Schematic diagram of the four morphologic types of pancreatic cystic lesions (Fan et al., Application of Contrast-Enhanced Ultrasound in Cystic Pancreatic Lesions Using a Simplified Classification Diagnostic Criterion. Biomed Res Int, 2015. p. 974621).

The classification standards by CEUS and MRI referred to the classification criteria summarized in the previous study of our center and MRI from the Sahani et al. [3] studies, respectively. Four types were classified: Type I are unilocular cysts without any septa or solid components, and the wall is thin and uniform. Type II are microcystic lesions, and the lesions consist of several microcysts ranging in size from a few millimeters to 2 centimeters. Type III are macrocystic lesions. There are less compartments in the lesion than in type II, and the maximum diameter of a compartment is often greater than 2 cm. Type IV are lesions with solid components, or the wall and septa are thickened irregularly (greater than 3 mm).  Figure 1 shows the schematic diagram. The diagnosis was based on the largest lesion when the patient had multiple cystic lesions.

### 2.5. Diagnostic Criteria of Pancreatic Cystic Lesions by CEUS and MRI

The lesions classified into type I by CEUS were considered simple cysts. Common simple cysts are pseudocysts, and other types included lymphoepithelial cysts and true cysts. The cysts contain pancreatic fluid and the wall is thin. There are no enhanced solid components inside.

Type II lesions were most likely considered serous cystic neoplasms (SCNs). SCNs are usually found in the head of the pancreas. Thin wall, microcysts, and lobe shape are the typical features. The maximum diameter of the microcysts is less than 2 cm.

Type III lesions were diagnosed as mucinous neoplasms (MCNs). The imaging features of MCNs are single cyst or less cysts with fine septa inside. There are usually thicker cyst wall and/or nodules (they are classified into type IV in this case). The enhancement of the wall and/or septa can be seen by CEUS or enhanced MRI.

Type IV lesions contain solid components. Common diseases include solid tumors, such as pancreatic carcinomas with cyst degeneration and solid pseudopapillary tumors (SPTs).

Particularly, if the lesion is communicated with the pancreatic duct, it is characteristic of intraductal papillary mucinous neoplasm (IPMN). Diagnostic criteria for common lesions of the pancreas by CEUS are shown in Table 1.

### 2.6. Statistical Analysis

Statistical analysis was performed using SPSS 23.0 statistical software. The measurement data were expressed as mean ± standard deviation; the kappa test was used to analyse the agreement between CEUS and enhanced MRI. The diagnostic value of classification by CEUS was analysed. The diagnostic sensitivity, specificity, accuracy, positive predictive value (PPV), and negative predictive value (NPV) of classification by CEUS for diagnosing different types of lesions were analysed. *P* < 0.05 was accepted as statistically significant.

## 3. Results

### 3.1. Classification Results of 84 Lesions by CEUS and MRI

The classification results of CEUS and MRI are shown in Table 2. Among the 84 cases, CEUS diagnosed 9 cases of type I, 24 cases of type II, 9 cases of type III, and 42 cases of type IV. Enhanced MRI diagnosis revealed 10 cases of type I, 25 cases of type II, 7 cases of type III, and 42 cases of type IV. The kappa value was 0.852 (95% CI: 0.76–0.95). The agreement of the two methods is significant. The results are shown in Table 2.

### 3.2. CEUS Classification Results of 45 Cases with Pathology Results

Among the 45 cases of pancreatic cystic lesions with pathology, 2 cases were classified into type I (2/45, 4.4%), 6 cases into type II (6/45, 13.3%), 5 cases into type III (5/45, 11.1%) and 32 cases into type IV (32/45, 71.1%). The diagnostic results of 45 cases are shown in Table 3.

### 3.3. Diagnostic Value of CEUS Classification

The diagnostic accuracy, sensitivity, specificity, PPV, and NPV of each type were analysed. The results are shown in Table 4.

### 3.4. Comparison of CEUS and MRI in the Diagnosis of 45 Cases

With pathological diagnosis as the gold standard, the accuracy of CEUS diagnosis was 75.56% (34/45), and the accuracy of MRI diagnosis was 80% (36/45). There was no significant difference between the two (*P*=0.687). The diagnosis results of both are shown in Table 5.

## 4. Discussion

Pancreatic cystic lesions can be roughly divided into nonneoplastic cystic lesions and neoplastic cystic lesions. Common diseases include pseudocysts, serous/mucinous cystic neoplasms/carcinoma, intraductal papillary mucinous neoplasms of the pancreas, and some solid lesions, such as pancreatic carcinoma and solid pseudopapillary tumors with cystic generation. Their histological origin, pathological features, and treatment are different. Biological behaviors range from benign to malignant; therefore, accurate imaging diagnosis plays an important role in guiding treatment and diagnosis. With high spatial and temporal resolution, CEUS can dynamically observe the blood flow perfusion and the internal fine structure of the lesion in real time. In recent years, it has been increasingly used to diagnose pancreatic cystic lesions. Relying on high soft tissue resolution, MRI is the main imaging method for diagnosing cystic lesions of the pancreas. However, it has certain limitations: its contraindications for examination and difficulty in observing the internal structure of small lesions. It has been reported that CEUS and MRI results of anatomical structures for the observation of solid lesions of the pancreas are comparable [4], and the results of our study are consistent with this.

Type I lesions are simple cystic lesions without septa or solid components. The lesions on CEUS showed no enhancement from the arterial phase to the parenchymal phase. The lesions on MRI showed a thin wall without septa or solid components. Cystic compoments inside showed a high signal on the T2-weighted images. For this type, CEUS and MRI have high consistency. The common disease of this type is pseudocyst. The pancreatic pseudocyst mostly occurs after acute and chronic pancreatitis or pancreatic injury. The inside of the cyst contains pancreatic juice and necrotic tissue, occasionally combined with hemorrhage and calcification. In this study, one patient was confirmed with pseudocyst by pathology. Because of internal hemorrhage deposition, CEUS revealed that there are internally enhanced solid components and misdiagnosed it as type IV, considering SPT. MRI was correctly diagnosed because of the high sensitivity of the DWI sequence to bleeding. The malignant rate of pseudocysts is extremely low because there is no solid component inside. When the cyst is less than 6 cm, conservative treatment or follow-up is often recommended. Endoscopic drainage or surgical treatment is selected when the cyst is larger than 6 cm or there is a high risk of bleeding/rupture. In this study, the sensitivity and positive predictive value of type I were low, considering the following factors: (1) The number of cases is small. Because follow-up rather than surgery/puncture is chosen for treatment of cystic lesions found in imaging examinations, it is difficult to obtain pathological results for these cases. (2) The simple cysts with pathological results included in this study were mostly rare pathological types, which made the diagnosis difficult.

In this study, 2 cases of lymphatic epithelial cysts were diagnosed incorrectly by MRI and CEUS. Lymphatic epithelial cyst is a rare nonneoplastic cystic lesion of the pancreas [5]. It has been reported to occur mostly in middle-aged men [5, 6], and the origin of histology is not clear at present. The cyst wall is composed of mature keratinized squamous epithelium, and the inner layer secretes keratinous protein, which causes a “cheese-like” change inside. Its imaging characteristics are not typical, and it can be composed of single or multiple rooms [7], which makes it challenging to distinguish it from other pancreatic cystic lesions. MRI and CEUS misdiagnosed the 2 cases as cystadenoma due to the enhanced solid components inside the lesion.

**Figure 2:**
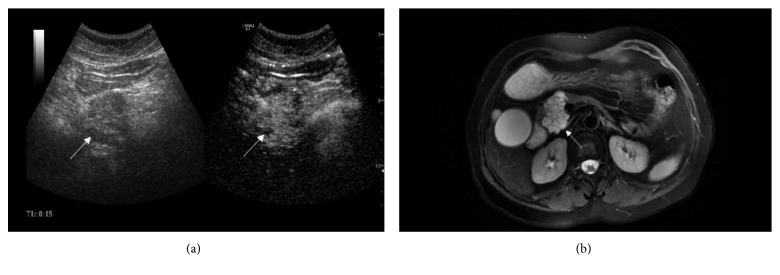
A 66-year-old woman with SCN in the pancreatic head. (a) CEUS shows the lesion is microcystic, the septa inside are enhanced, and microcysts are nonenhanced. (b) On T2-weighted MR image, the multiple septa show low signal.

The type II lesion is composed of a plurality of microcysts, and the maximum diameter of each microcyst is no more than 2 cm. The most common disease of this type is SCNs. SCNs are often observed in the head of the pancreas. The typical feature is that the edge of the lesion is slightly lobulated, the inside is a microcapsule-like honeycomb, and the wall is thin [8]. Nougaret et al. [9] reported that the radial scar of the center may be the characteristic performance. SCNs were considered benign and rarely malignant [10, 11], so the guidelines [12] recommended regular follow-up for these lesions. Surgical treatment should be performed when there is oppression or malignant tendency. Choi et al. [13] hold the view that a small number of SCNs are single cyst, which were difficult to distinguish from large cystic lesions such as mucinous cytic neoplasms and pseudocysts [14]. At this time, it may be helpful for diagnosis that the lesion located at the head of the pancreas and its fractal contour. The final diagnosis depends on pathological diagnosis. Five SCNs in this study were all microcystic. CEUS showed that the multiple septa were obviously enhanced in the arterial phase, and most of the rest was not enhanced (Figure 2).

**Figure 3:**
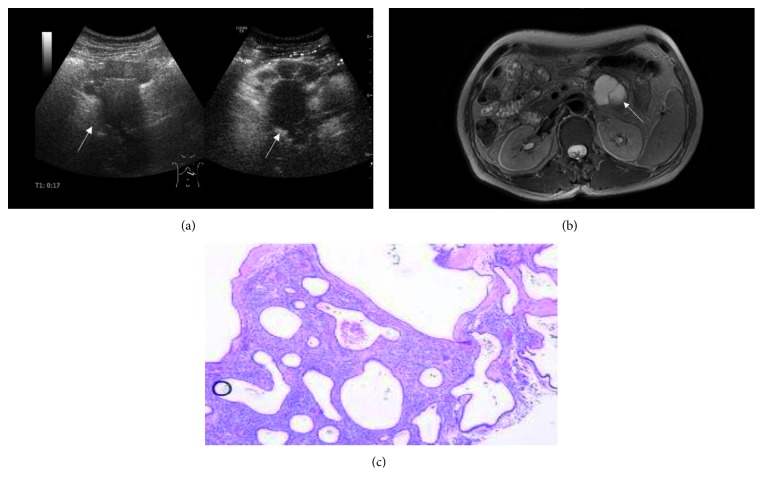
A 60-year-old woman with MCN in the pancreatic tail. (a) CEUS demonstrates a lesion with less septa inside (pointed by the arrow). (b) T2-weighted MR image shows the septa are low signal, and the liquid components are high signal (pointed by the arrow). (c) Photomicrograph shows that the lesion is composed of some macrocysts (H&E stain, ×200).

The main feature of type III lesions is that there are fewer cysts, and the maximum diameter of a single cyst is often greater than 2 cm. The common disease of this type is MCNs. MCNs are often seen in middle-aged women and mostly in the tail of the pancreas [15] (5 cases of MCNs in this study observed in the tail of the pancreas). Because of the malignant risks of MCNs [15, 16], the guideline [12] recommended that patients diagnosed with MCNs should accept surgery, especially when there are nodules or the size is greater than 3 cm. Its typical imaging character was single or small cysts, mostly without lobes, and there are thicker cyst wall and fine septa, occasionally with nodules on the wall (this case was classified as type IV). The enhancement of the wall and septa can be seen in both CEUS and MRI (Figure 3). In this study, type III was considered as the diagnostic criteria for MCNs. The diagnostic accuracy and specificity were high. However, one MCN lesion showed a single cyst; CEUS showed no enhanced septum and misdiagnosed it as a pseudocyst. Many articles [17, 18] believed that single-cyst MCN was difficult to differentiate from pseudocyst and single-cyst SCN, which depended on pathological results finally.

Common type IV diseases were pancreatic carcinoma with cyst generation, malignant cystic adenoma, IPMN, SPT, and the like. These diseases are relatively easy to classify because of their enhanced solid components. Pancreatic carcinoma is a common malignant tumor of the pancreas, usually with liquefactive necrosis. It has a typical enhancement feature that the lesion is enhanced later than the pancreatic parenchyma. At the same time, with its invasive biological characteristics, diagnosis is not difficult. This study included an elderly male patient with pancreatic carcinoma; due to enhancement with mass in the arterial phase and a history of penile cancer, we considered neuroendocrine tumors (tumors with abundant blood supply) first, and penile cancer metastasis was not excluded by analyzing CEUS video. Other than this, the remaining pancreatic carcinoma cases were diagnosed accurately.

IPMN is a tumor that originates from the epithelial duct of the pancreas and secretes mucin. According to the origin of the lesion, IPMN can be divided into the main pancreatic duct (MD-IPMN), the branched pancreatic duct (BD-IPMN), and mixed-type IPMN (MT-IPMN) [19]. MD-IPMN and MT-IPMN are mostly invasive growth with high malignant risk. Therefore, surgical treatment should be preferred once diagnosed. Because BD-IPMN does not invade the main pancreatic duct and has a low malignancy risk, the guidelines recommend that lesions with diameter <3 cm be followed up. But surgical treatment is recommended in the case of lesions with wall nodules [12]. Imaging characteristics of IPMN: the pancreatic duct is limited or diffusely dilated, and there may be enhanced nodules in the lesion. MRI is the preferred follow-up imaging modality [12]. CEUS has been reported to have a similar resolution to the enhanced MRI, and therefore may be an effective imaging method. The primary purpose of imaging diagnosis is to confirm whether the lesion is connected to the main pancreatic duct, and whether there are solid nodules in the lesion.

In this study, 1 case of IPMN with invasive carcinoma was misdiagnosed as SPT by CEUS and MRI because the junction between the lesion and the pancreatic duct was not obvious and the lesion capsule was ring-shaped and enhanced in both CEUS and MRI. We thought the misdiagnosis was possibly related to the nonspecific manifestation of the lesion and the complex internal structure.

**Figure 4:**
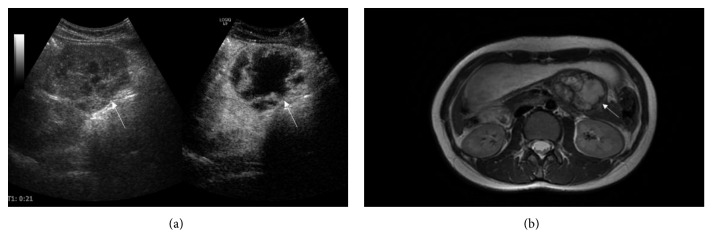
A 17-year-old woman with a SPT in the pancreatic tail. (a) CEUS shows the ring-shaped enhancement of the tumor and the solid components are enhanced separately. (b) Enhanced T2-weighted MR image shows the lesion with capsule, which is progressively enhanced.

SPTs are rare in clinical practice and most patients are young women. In this study, 5 of the 6 patients who were diagnosed with SPTs were women. SPTs are generally large in volume and have a complete fibrous capsule (Figure 4). It consists of a solid part, a pseudo-nipple part, and a transitional part of the two. The typical performance of MRI is the high signal in the cystic part of the lesion on T2-weighted images and the internal hemorrhage showed high signal on DWI. Progressive enhancement was another characteristic [20]. The CEUS of SPTs is characterized by ring-shaped enhancement of the capsule in the arterial phase, and the solid component is iso-enhanced [21]. Calcification is often seen as well.

Common diseases of types I and II are benign and are often followed up for clinical observation. Evaluating the type preoperatively can help assess the malignant risk and avoid unnecessary surgery. Types III and IV have enhanced solid components, and common diseases are malignant or have high malignant tendency; therefore, these two types should be paid attention to and patients with these conditions should accept surgical treatment mostly.

## 5. Conclusion

The imaging features of pancreatic cystic lesions are easily confused. Combined with CEUS and anatomical features, the classification can indicate the nature of the lesions, benign or malignant, and help provide some information for diagnosis and treatment. CEUS classification is highly consistent with that by MRI in pancreatic cystic lesions. CEUS is simple and nonradiative, has short-term repeatability, and hence can be used as an effective diagnostic and follow-up method.

## 6. Limitations

This study also has certain limitations. Patients with types I and II diseases often choose follow-up, which limits the sample size of the study, especially in type I. According to clinical experience, CEUS and MRI have better diagnostic efficacy for simple cystic lesions. As the sample size increases, the diagnostic efficacy index of type I may increase.

## Figures and Tables

**Table 1:** CEUS diagnostic criteria for common pancreatic cystic lesions.

Diseases	CEUS criteria	MRI criteria
Pancreatic carcinoma with cyst degeneration	In the arterial phase, lesions enhance later than the pancreatic parenchyma and is hypoenhanced, with signs of metastasis	In the arterial phase, lesions enhance later than the pancreatic parenchyma and is hypoenhanced, with signs of metastasis

SPT	The lesion is ring-shaped and enhanced in the arterial phase, the solid parts can be enhanced, and there are cystic nonenhanced parts inside	The lesion is ring-shaped and enhanced in the arterial phase, and there are cystic parts or calcification or bleeding inside

IPMN	Polycystic lesions are connected to the pancreatic duct, and solid nodules can be seen inside	Polycystic lesions are connected to the pancreatic duct, and solid nodules can be seen inside

SCN: serous cystic neoplasm; MCN: mucinous cystic neoplasm; SPT: solid pseudopapillary tumor; IPMN: intraductal papillary mucinous neoplasm.

**Table 2:** Comparison of CEUS and MRI classification of 84 cases.

	CEUS					
		I	II	III	IV	Total
MRI	I	7	2	1	0	10
	II	1	22	0	2	25
	III	0	0	7	0	7
	IV	1	0	1	40	42
	Total	9	24	9	42	84

**Table 3:** CEUS classification of 45 cases with pathological results.

Diagnosis	CEUS classification				
	I	II	III	IV	Total
Simple cyst	1	1	1	1	4
SCN	0	5	0	0	5
MCN	1	0	3	1	5
IPMN	0	0	1	7	8
SPT	0	0	0	6	6
Ca	0	0	0	16	16
Pancreatitis	0	0	0	1	1
Total	2	6	5	32	45

SCN: serous cystic neoplasm; MCN: mucinous cystic neoplasm;SPT: solid pseudopapillary tumor; IPMN: intraductal papillary mucinous neoplasm; Ca: pancreatic carcinoma.

**Table 4:** Diagnostic value of CEUS classification on 45 lesions.

Type	Accuracy	Sensitivity	Specificity	PPV	NPV
I	91.1% (41/45)	25.0% (1/4)	97.6% (40/41)	50.0% (1/2)	93.0% (40/43)
II	95.6% (43/45)	83.3% (5/6)	97.4% (38/39)	83.3% (5/6)	97.4% (38/39)
III	93.3% (42/45)	66.7% (4/6)	97.4% (38/39)	80.0% (4/5)	95.0% (38/40)
IV	88.9% (40/45)	96.6% (28/29)	75.0% (12/16)	87.5% (28/32)	92.3% (12/13)

CEUS: contrast-enhanced ultrasound; PPV: positive predictive value; NPV: negative predictive value.

**Table 5:** Comparison of diagnostic accuracy of 45 cases of CEUS and MRI.

	Diagnosis	Pseudocyst	Lymphatic epithelial cyst	Serous cystic neoplasms	Mucinous neoplasms	Mucinous intraductal expansion	Intraductal papillary mucinous neoplasm	Pancreatic carcinoma	Solid pseudopapillary tumor	Pancreatitis
CEUS	Correct	0	0	5	5	0	6	13	5	0
	Misdiagno-sis	1	2	0	2	1	2	1	1	1

MRI	Correct	1	0	5	5	0	7	13	5	0
	Misdiagno-sis	0	2	0	2	1	1	1	1	1

## Data Availability

The data used to support the findings of this study are unavailable because our study is a clinical research and all data are related to patients' privacy.
